# Research on College English Classroom Teaching Model Based on Adaptive Genetic Algorithm

**DOI:** 10.1155/2022/9527070

**Published:** 2022-03-15

**Authors:** Zhiling Yang

**Affiliations:** ^1^School of Foreign Studies, Wenzhou University, Wenzhou, Zhejiang 325035, China; ^2^College of Industrial Education, Technological University of the Philippines, Manila, Philippines

## Abstract

While college English teaching is steadily changing from static knowledge transfer to dynamic language ability development, classroom activities centered on language application are becoming more and more important in cultivating students' language application ability. In recent years, education has been paid more and more attention, the scale of university education has gradually expanded, the professional categories have become more and more complete, the curriculum has become larger and larger, and the number of students has grown by leaps and bounds. The teaching resources (teachers, classrooms, teaching equipment, etc.) and the workload of English teachers are increasing. In order to effectively improve the efficiency of college English teaching, the paper proposes to apply genetic algorithms to the actual English course scheduling problem in colleges, taking into account all the various hardware and software constraints and the expected course scheduling goals, so as to provide a clear and concise solution to the course scheduling problem plan (parallel search for optimal scheduling) and the design and coding structure of each genetic operator. Furthermore, this study creates a genetic algorithm-based English social platform and examines the design aspects of dynamic teaching models and classroom activities of college English students in the context of this paper.

## 1. Introduction

A major source of worry for university English instructors, as well as many language scholars and students, has been how to enhance the quality of teaching and learning of English at university and how to increase the efficacy of language learning. As a consequence, in recent years, new reform ideas and efforts in the field of language education have sprung up [1, 2]. As language education has progressed over the years, it has moved away from the conventional static paradigm toward a dynamic one, and the teaching process is no longer limited to the typical “teacher talks at the podium while pupils sit in the classroom and listen.” Instead of just imparting content, the goal of language instruction nowadays is for pupils to master language abilities rather than simply comprehending it. According to the actual teaching process and its consequences, the teacher's lecture at the podium does not compare well to the activities conducted in the classroom [3]. It is students who are the primary source of language acquisition, and it is only through suitable classroom activities that students' engagement and enthusiasm in the learning process can be mobilized [4]. As a result, when it comes to classroom instruction, teachers should not limit themselves to traditional methods of instruction, but should instead take the initiative to create engaging classroom situations, create a relaxed and positive teaching environment, encourage students to actively participate in classroom discussion, and stimulate their learning enthusiasm [5].

The archetypal picture of traditional teaching is that of a teacher standing on a stage with a textbook in front of him or her, speaking orally while students sit in their seats, listening absentmindedly. First and foremost, it is critical to emphasize that the conventional teaching style is not inherently ineffective. Some courses or challenging areas of language acquisition need a methodical and concentrated presentation at the lectern in order for students to gain a great deal of information and experience in a short period of time. Traditional teaching, on the other hand, has one major drawback: most pupils have difficulties sustaining a high level of focus for extended periods of time. Student attention spans are very restricted if they are passive receivers of information, and they are often preoccupied with taking notes and keeping up with the teacher's ideas that they are unable to engage in active thinking or participate in the predetermined rhythm of teaching. In actuality, the great majority of students are passive learners who have no visible motivation to study and no preconceived knowledge of the classroom or the curriculum, despite the fact that they are in class [6–8]. Furthermore, for students who have studied classroom knowledge, they often have the dilemma of doing nothing in the classroom, believing that “I know everything the instructor stated,” as is the case in the conventional teaching form of instruction. In this way, traditional teaching is a contradiction: if students have studied in advance, they will find the teacher's lectures boring; if students have not studied in advance, the teacher's lectures may be new to them, but they will be too busy to think actively about the new knowledge, or they will be unable to follow the teacher's lecture rhythm, and as a result, they will be unable to concentrate.

This is the difficulty with conventional methods of instruction. The reason for this is because the conventional teaching model is a static teaching process in which the instructor prepares the lesson in advance and then teaches it to the students in class, therefore completing the transfer of information or skills from one group of students to another. The primary goal of lesson preparation is to organize information and theory in a clear and understandable manner, preferably with accompanying practice problems. The dynamic teaching approach, on the other hand, necessitates the preparation of lessons beyond just sorting out facts and providing activities. Students' thinking is stimulated, and their knowledge and skills are solidified. The most important characteristic of dynamic teaching is that teachers must transform learning contents into actionable teaching activities and arrange appropriate classroom activities to either stimulate students' thinking or solidify students' knowledge and skills. The manner in which instructors modify the content of their lessons is critical, and the outcome of teachers' transformation of knowledge points is mirrored in classroom activities [9, 10].

Today, the Internet not only plays an optimal and integrated role in the allocation of production factors, but it has also been integrated into the basic requirements for teaching public English courses in schools promulgated by the Ministry of Education, emphasizing the importance of actively introducing and using computer and network technologies, using information technology to reasonably optimize English teaching conditions in schools and enhancing students' *s* learning outcomes in public English courses. The employment of blended learning modes and “Internet Plus” technologies in public English education may, at the same time, satisfy the demands of English education development.

In the new millennium, “Internet +” is being applied to all sectors of the economy and society, which is assisting in the improvement of real-world creativity and productivity, as well as the formation of a new type of economic development based on the Internet. In the implementation of the “Internet +” action plan, cloud computing, big data, and the Internet of Things will serve as representatives of new information technology and modern manufacturing, service integration, comprehensive optimization, and the growth of new industries, as well as to create new industrial growth points and a favorable environment for mass entrepreneurship and innovation. “Internet +” action plan: it may also combine the development features of industrial intelligence in order to increase the velocity of economic growth as well as the efficiency with which the national economy develops.

It is a hybrid kind of online + offline education that incorporates the benefits of both online and conventional teaching. Blended public English teaching may be described as follows: additionally, this organic blend of multiple styles of instructional structure has the potential to engage students in deep and varied learning. However, the ultimate goal of public English instruction in blended learning is not to make use of the online platform, but rather to enable students to participate more fully in blended learning activities by carrying out a variety of teaching activities and increasing the depth of student learning. There are four major paths that learners can take in blended learning: the first is the process of active participation on their part; the second is the process of gradual knowledge accumulation; the third is the method of communication used during various types of learning and inquiry; and the fourth is the provision of accurate and timely external support environments and activities on their part for learners [11].

At the end of the twentieth century, the exploration and practice of various scheduling modes began in China, resulting in a variety of scheduling systems in universities today, such as resource-based human-computer interaction scheduling systems and FFD algorithm-based scheduling systems in universities. Increasingly in recent years, as a result of the fast development of swarm intelligence algorithms, swarm intelligence algorithms have been used to the challenge of scheduling classes at colleges [6]. Many issues are difficult to take into consideration when manual scheduling is used, and to a certain degree, just the regular providing of English courses can be ensured if difficulties such as resource optimization are not taken into consideration. Due to the fact that adaptive genetic algorithms can take into account all of the soft and hard restrictions of class scheduling, they can solve the scheduling issue in a cost-effective manner, and they are simple to implement programmatically; they may be utilized for intelligent class scheduling. Because genetic algorithms have the advantages of a unique population search strategy, flexible and diverse coding, and strong parameter robustness, they are well suited for solving multiobjective and multiconstraint scheduling optimization problems. As a result, genetic algorithms have been selected for English scheduling because of their unique population search strategy, flexible and diverse coding, and strong parameter robustness [12, 13].

Although some researchers have made significant contributions to the solution of the English scheduling problem using an improved genetic algorithm, there are still some issues to be addressed, such as the requirement that all individuals in the initial population be conflict-free, which is problematic in the real world of English teaching, where teaching resources are limited, teachers may have to teach multiple classes under multiple grades, and a classroom may have to schedule multiple classes, and the randomly generated genetic algorithm is not a good solution. In addition, the design of the fitness function is not sufficiently adaptable to changing circumstances. In actuality, the instructor will impose certain limits on the school environment, but the fitness function in this study only takes into account a few preset constraints. As a result, the scheduling system is more difficult to maintain in the future. Certain current scheduling systems, like KDDI's intelligent scheduling system, are also lacking in effective solutions for the complicated and specific demands of some schools, such as long and short courses, tutor classes, and school-based course scheduling, among other things [14, 15].

Using these issues as a starting point, we develop a solution to the English scheduling problem, including a method to automatically divide the teaching classes without taking into account conflicts in the class schedule when generating the initial population, thereby removing the requirement that individuals in the initial population must be feasible solutions; in addition, we introduce the concept of gene editing into the variation operator, which allows us to automatically locate and eliminate on-target candidates [16]. The algorithm's performance in practical class scheduling issues is shown by the results of the practical verification phase. Aside from that, this study investigates the teaching methodology used in a college English classroom and develops a social platform for learning English that is powered by a genetic algorithm [17].

## 2. Genetic Algorithm

**Figure 1:**
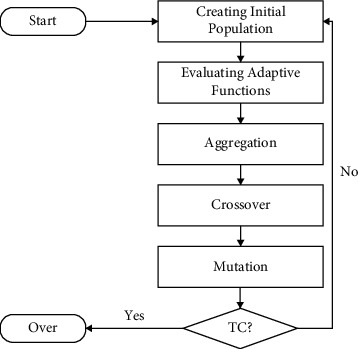
Genetic algorithm flow chart.

The genetic algorithm (GA) is a meta-heuristic tool that may be used to find the most optimal solutions. A cornerstone in the domains of data mining, medicine, and data science, and Darwin's evolutionary-based genetic algorithm has proved to be a breakthrough in the field of genetic algorithms [18]. An adaptive process, such as the genetic algorithm shown in Figure 1, is demonstrated by the genetic algorithm. Figure 1 depicts a flow diagram of the genetic algorithm's implementation throughout its development. Genetic algorithm has good imitative properties and also has the function of artificial intelligence and has good application value in the teaching process.

**Figure 2:**
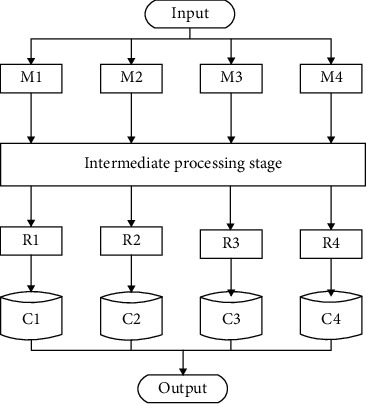
Genetic algorithms in Hadoop MapReduce framework.

As seen in Figure 1, the genetic algorithm consists of five phases, each of which includes five operations such as initialization, assessment of the fitness function, aggregation, crossover, and mutation. Genetic algorithms are defined differently for each task, and they handle three types of problems: (1) how should the population be initialized? (2) How should the fitness function be evaluated? (3) What is the outcome of the aggregation? The adaptive function defines the labeling of other population values, and the selection technique is based on the notion of “pick the best and reject the rest.” Molecular genetic operators such as crossover and mutation are both used in genetic research. Crossover operators are responsible for swapping pairs or single genes in a chosen population, while mutation is the new information created by modifying the crossover operation. According to the clustering specification, a single tweet represents a population, and each chromosome represents a cluster grouping [19, 20]. A gene is an object that has clustered allele values, and each tweet is grouped into the same cluster as a chromosomal gene that has the same allele value. As two concepts, developers must describe chromosomes as a collection of arrays and may define genetic operators as a set of genetic operators. One is the fission of allele values, and the other is the fusion of allele values in order to unite clusters or strengthen clustering. As shown in Figure 2, the distribution of social platform data uses GA and working on the Hadoop MapReduce framework, where the social platform data is divided equally into mappers and illustrates the initialization, adaptive function, and selection process to submit the data to the reducer according to the fitness function cross pursuit and mutation, where Mi, Ri, and Ci denote the form of transformation at different stages [21].

## 3. Method Design

The core task of the scheduling problem is to arrange courses, classes, instructors, and classrooms in a nonconflicting manner and to ensure that the constraints set by the instructor are met and that the results are optimal or approximately optimal.

In (1), we see that the mathematical model of the scheduling issue is stated as follows: the set of courses (SC), the set of classes (CC), the set of instructors (TC), the set of classrooms (RC), and the set of time slots (PC), as illustrated in Figure 1.

(1) $$\begin{array}{c} SC=\left\{s_{1},s_{2},\ldots ,s_{s}\right\},\\ CC=\left\{c_{1},c_{2},\ldots ,c_{s}\right\},\\ TC=\left\{t_{1},t_{2},\ldots ,t_{s}\right\},\\ RC=\left\{r_{1},r_{2},\ldots ,r_{s}\right\},\\ PC=\left\{p_{1},p_{2},\ldots ,p_{s}\right\}. \end{array}$$

We must first determine the class schedule for each grade level, then determine the relationship between course, class, teacher, and classroom, i.e., a specific class should be taught by a specific teacher in a specific classroom, find a pair of lecture time to satisfy the constraints, and ensure that teaching resources do not conflict with one another, which is the process of solving the scheduling problem, and they form a set of lectures, *D*={*s*, *c*, *t*, *r*, *p*}.

### 3.1. Binding Conditions

It is necessary to consider not only whether the elements of the class schedule are reasonable in terms of time and space but also whether they comply with certain constraints established by the teaching staff in order to ensure that the elements of the schedule do not conflict with one another, that the teaching resources can be combined and allocated as efficiently as possible, and that teaching work can be carried out normally. In terms of limits, they may be classified into two categories: harsh restrictions and soft restrictions.

In the scheduling process, the hard constraints are the requirements that must be met, and only when the schedule satisfies the hard constraints can we be certain that the scheduled resources do not clash with one another, as shown in the following diagram.

(1) The same class can only be scheduled for a maximum of one course in the same teaching period.
  (2) $$\begin{array}{c} \sum_{m=1}^{CC}\sum_{i=1}^{PC}\sum_{j=1}^{SC}c_{m}p_{i}s_{j}t_{n}r_{k}\leq 1. \end{array}$$
(2) The same classroom can only be scheduled for a maximum of one course in the same class period.
  (3) $$\begin{array}{c} \sum_{k=1}^{RC}\sum_{i=1}^{PC}\sum_{j=1}^{SC}c_{m}p_{i}s_{j}t_{n}r_{k}\leq 1. \end{array}$$
(3) The same teacher can only schedule a maximum of one course in the same teaching period.
  (4) $$\begin{array}{c} \sum_{n=1}^{TC}\sum_{i=1}^{PC}\sum_{j=1}^{SC}c_{m}p_{i}s_{j}t_{n}r_{k}\leq 1. \end{array}$$
(4) Teachers with mutually exclusive rules cannot teach classes in the same period; we have
  (5) $$\begin{array}{c} p_{1}\neq p_{2}. \end{array}$$

“Soft constraints” are restrictions established by the faculty before scheduling that are not required to be satisfied during the scheduling process but have a substantial influence on the rationale of the schedule and the happiness of the users who utilize the schedule.

According to these rules, the extent to which a schedule adheres to them affects the schedule's performance in the following ways:

1. The weekly schedule of the same course should be spread out as much as possible.
2. When a course is scheduled to have priority, the course should be scheduled in the session that has the highest priority, e.g., the main course should be scheduled in the morning.
3. The instructor's consecutive schedule is the maximum number of consecutive sessions.
4. Some classes are not scheduled immediately after others, and so on.

### 3.2. Fitness Function

The fitness of an individual is a significant indication in genetic algorithms since it describes the person's strengths and flaws (class schedule). Individuals are picked depending on their fitness throughout the iterative phase of the algorithm. The construction of the fitness function decides whether or not the genetic algorithm is capable of obtaining the global optimum solution. In order to account for the unique characteristics of the high school schedule, the fitness function in this paper takes into account two aspects, namely, the priority of the courses and the uniformity of the course schedule, and incorporates a penalty measure to reduce the fitness of individuals who violate both hard and soft constraints.

(1) Section priority is a criterion for evaluating the merits of a course scheduling strategy. The instructor may establish the priority of each class before scheduling it; for example, the primary class may be planned in the morning, and the greater the number of courses in the timetable that fits the priority rule, the higher the priority of the class will be set by the instructor. According to the definition in this work, the section priority of the scheduling scheme is represented by *f*_1_, and the related formula is
  (6) $$\begin{array}{c} f_{1}=\delta^{\text{vios}}, \end{array}$$
where *δ* denotes the influence factor of course section priority and is generally taken as 0.9–0.99, which indicates the number of times the course arrangement in the scheduling scheme violates the section priority rule, and the higher the number of violations, the smaller the value of *f*_1_.
(2) Course uniformity is a measure of how evenly the courses in a class are distributed over the course of a 1-week period, expressed as *b*_*s*_. Each course should be distributed as evenly as possible throughout the week to avoid over-concentration of a course in a certain period of the week.
First, consider the uniformity of *b*_*s*_ for 1 course in 1 class, which is calculated as
  (7) $$\begin{array}{c} b_{s}=\frac{1}{1+1/D\sum_{i=1}^{D}\left|p_{i}-p^{\prime }\right|}. \end{array}$$
Then is the uniformity of course scheduling for a class, denoted by *ρ*_*c*_, which is calculated as *ρ*_*c*_=1/*S*∑_*s*=1_^*S*^*b*_*s*_ (7).
In (6) and (7), *D* is the number of days per week, S is the number of courses taught in the class, *p*_*i*_ is the number of hours scheduled for the course on that day, *p*′ is the standard number of hours per day for the course, and
  (8) $$\begin{array}{c} p^{\prime }=\frac{p_{\text{period}}}{d} \end{array}$$
where *p*_period_ denotes the number of hours per week of the course, and *d* is the number of days per week of the course, and in this paper, we take *d* = 5.
In this paper, we define *f*_2_ to represent the uniformity of course scheduling for all classes, and the larger the value of *f*_2_, the better the scheduling solution, which is calculated as
  (9) $$\begin{array}{c} f_{2}=\frac{1}{C}\sum_{c=1}^{C}\rho_{c}, \end{array}$$
where C denotes the number of classes.
(3) If the schedule breaches the hard and soft restrictions, it is penalised by reducing the fitness by determining whether or not the schedule violates the constraints. The greater the number of infractions, the greater the degree to which adaptation has been compromised. When the punishment degree is indicated by *β*, it is followed by the matching equation.
  (10) $$\begin{array}{c} \beta =\alpha_{1}^{\text{hard\_vios}}\cdot \alpha_{2}^{\text{soft\_vios}}, \end{array}$$

where *α*_1_ and *α*_2_ are penalty factors, and *α*_1_ < *α*_2_ < 1 (the priority is to eliminate the conflict of hard constraint), hard_vios is the number of times an individual violates hard constraints, and soft_vios is the number of times an individual violates soft constraints. The values of *α*_1_ and *α*_2_ have a significant impact on the evolutionary effect. A small value will result in a large jump in fitness and a small impact of other factors on the overall fitness, while a large value will result in a small penalty on the fitness value.

In summary, the fitness function in this paper is given by

(11) $$\begin{array}{c} f=\left(\omega_{1}\cdot f_{1}+\omega_{2}\cdot f_{2}\right)\cdot \beta \end{array}$$

where *ω*_1_ and *ω*_2_ are weights, and *ω*_1_+*ω*_2_=1.

### 3.3. Genetic Algorithm Operation Operator Design

#### 3.3.1. Selection Operator

The selection operation simulates the phenomenon of survival of the fittest in nature. The algorithm selects some individuals to produce the next generation of individuals, while the rest are eliminated.

The selection operation in this paper combines the best retention strategy and roulette algorithm. To avoid that the best individuals in each generation are destroyed in later genetic operations, they are first copied into the next generation, and then the remaining individuals are selected using the roulette algorithm. The higher the fitness of an individual, the higher the probability that its genes will be passed on to the next generation.

Using the roulette algorithm, the probability of an individual being selected is the proportion of the individual's fitness to the sum of the fitnesses of all individuals in the population, proportional to the value of the individual's fitness.

#### 3.3.2. Crossover Operator

To generate a new human, a crossover operation is a procedure in which two people exchange part of their genes with each other according to a certain crossover probability. When running genetic algorithms, the crossover operation is critical since it is the most effective technique of creating new people throughout the course of the algorithm's execution. When it comes to strengthening the algorithm's capacity to search globally, crossover operations are critical steps.

The crossover approach is described as follows in this paper: there are two of them. Using a roulette wheel algorithm based on individual adaptation, Individual 1 and Individual 2 are selected, and one grade is chosen at random from among the gene objects of all classes under that grade in each of the two individuals. The gene objects of these classes involved in each individual are sorted out separately, and the gene objects of these classes involved in each of the two individuals are swapped.

#### 3.3.3. The Operator for Mutation

Using the mutation operation, you can imitate the mutation of genes that occur during the evolution of natural species. You may also replace certain genes in the chromosome with other genes based on a modest mutation chance to produce a new person. When a genetic algorithm is being executed, the mutation operation is used to produce new people as an auxiliary approach. This method increases the local search capabilities of the algorithm and guarantees that there is a variety of individuals inside the population.

To automatically detect conflicts and resolve them with a given probability, we add the concept of gene editing into the mutation algorithm in this study.

First, a class is randomly picked, and then all gene objects associated with the class are acquired from the people who will be changed in the following steps:

The first step determines whether there is a conflict between the set of gene objects obtained in the first step, which means whether the class schedule violates the hard constraint; if there is a conflict, the third step is executed with a certain probability; otherwise, the fifth step is executed immediately.

To complete the third step, first determine whether there is a conflict in the class schedule (2 or more classes are scheduled in the same class period); if there is, only 1 class will be reserved in this period, and the rest of the courses will be arranged in the free period (because the number of classes in a week in the same class is fixed, if there is 1 period with multiple classes, then there must be no classes scheduled in the free period); and then choose from *x* (x−1) from the conflicting Ge classes. The fourth step is carried out if there is no conflict in the class; otherwise, the current variant is exited.

Fourth, we determine whether there is a conflict in the schedule of the teacher and/or the classroom (2 or more classes scheduled by the same teacher or classroom in a given teaching period), and if there is a conflict, we randomly select a session from the schedule of the class and swap it with the conflicting session, for example, we randomly select a gene object Gene 1 and swap it with the arrangeTime property of a conflicting gene object Gene 2's arrangeTime property of a conflicting gene object Gene. When you have completed the fourth stage, you should save the modified persons and exit the mutation procedure.

Five steps later, choose two random course sessions in the class and switch them, i.e., choose two gene objects at random and exchange their arrangeTime attributes, and then repeat the process with the remaining course sessions.

Remember that only genes belonging to the same course may be switched during the mutation procedure. Because combination courses are scheduled in multiple classes at the same time, combination courses are swapped with other combination courses only; long-time courses are scheduled for 1.5 class periods, so long-time courses are swapped with other long-time courses only; regular-time nonwalking courses are scheduled for 1 class period, so courses of this type are swapped with courses of this type only; regular-time walking courses are scheduled for 1 class period, so courses of this type are swapped with courses of this type only.

## 4. Experimental Test Analysis

### 4.1. Experimentation Data and Experimental Environment

It is based on the curriculum of Shanghai East China Middle School, and the size of instruction and limits are introduced depending on the demand that is seen throughout the trial. The experimental data consist of 24 courses, 30 teaching areas, 13 topics, 118 instructors, and 40 teaching times per week (8 classes per day and 5 days per week) and a variety of scheduling limitations throughout the course of a full year of testing.

Linux is being used as the experimental environment.

### 4.2. Configuration of the Parameters

It is possible that how the parameters are chosen in a genetic algorithm will have a substantial influence on the overall performance of the algorithm. The following are the specifics:

1. The size of the population has a significant impact on the efficiency and convergence of the algorithm, as discussed before. Increasing the size makes it simpler to reach the local optimum solution; decreasing the size makes it harder to get the local optimal solution and increases the computational speed of the genetic algorithm. Increasing the size also increases the amount of memory required for the calculation. As a result, the population size should be limited to 500 people.
2. The number of iterations of the genetic algorithm has a significant impact on the performance of search results as well as the length of time it takes to complete a search. A genetic algorithm's execution time will be significantly increased if the number of iterations is too little; if the number of iterations is too big, the execution time will be significantly increased. This is the number of genetic iterations that will be used, which is set at 3000.
3. The crossover probability determines the frequency with which crossover operations are performed. In general, the larger the crossover probability, the faster the generation of new individuals will be. However, a crossover probability that is too large can easily eliminate individuals who have high fitness in the population; a crossover probability that is too small can cause the search process of the algorithm to slow down, or even stall. The likelihood of a crossing is set at 0.6 percent.
4. The frequency with which variation operations are performed is controlled by the variation probability. It is possible to generate more new individuals if the variation rate is set too low; however, it is easy to destroy the good genes in the individuals if the variation rate is set too high, and the algorithm degenerates into a purely random search algorithm if the variation rate is set too low or too high. The likelihood of variance has been set at 0.1.
5. Additional parameters: course portions are listed in the fitness function of this method, as are the influence factor and punishment factor of each part. The penalty factor XXXXXXX is a parameter that, when included in the schedule, lowers individual adaptability as a result of the existence of both hard and soft restrictions. It is possible that if the value is too little, the adaptation will be significantly decreased; this will result in just a minor influence of other parameters in terms of total adaptation; on the other hand, if the value is too great, the effect on adaptation will be negligible. Furthermore, because of the necessity to avoid hard conflicts, the value of the penalty factor for hard constraints is less than the value of the penalty factor for soft constraints, which is set to 0.88 and 0.94 in this study, respectively.

### 4.3. Experimental Results

**Figure 3:**
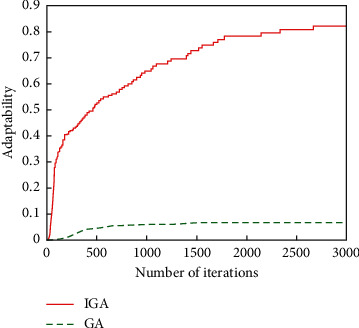
Comparison of the effects of the two methods.

The genetic algorithm (GA) without gene editing and the improved genetic algorithm (IGA) proposed in this paper were used first, and the comparison of the evolutionary effects of the genetic algorithm for the two experiments is shown in Figure 3.

From Figure 3, it can be seen that the fitness of the improved algorithm IGA increases rapidly in the first 1,700 generations of the population, and the fitness of the later generations tends to be stable, because the GA method may have more conflicts that are difficult to eliminate, so the fitness increases slowly, so the evolutionary effect of IGA is significantly better than that of GA.

### 4.4. Genetic Algorithm-Based Social Platform for English

The algorithm designed in this paper is as follows.

1. Initialization of Population(P)
2. While (P has next_value)
3. Map_reduce = startmap_reduce_job 4.*P* = map_reduce(p)
4. *P* = map_reduce(p)
5. Save results into intermediate stage using GA
6. The Reducer will start clustering according to pair save into HDFS
7. Step 3 repeats till all intermediate stage has values
8. HDFS writes all clusters into one file

The algorithm is executed in Mapreduce, which executes the algorithm as follows.

1. Splitting of sequence file to mappers
2. Initiate selection_strategy (roulette wheel)
3. Mappers evaluate Fitness function
4. Generate new rulespair
5. Repeat until fitnessop = new arrayList(evol_op)
6. List(evol_op)op = new arrayList(evol_op)
7. op.add(new Crossover(p))
8. op.add(new Mutation(p))
9. Evol_Pipeline pipeline = newEvolutionPipelin(op); start new_selection_strategy and repeat the process until the threshold value is reached. The reason for using the proportional selection operator method as a selection process is the probability-based selection method where the selection criterion is to choose the best first to save time and efficiency.

## 5. Conclusion

In the context of professional accreditation of engineering education, the innovation of traditional experimental scheduling mode is in line with the trend of social development and is also an inevitable trend of teaching development. This paper discusses the main influencing factors, constraints, and solution objectives in the scheduling problem of university and college in a more practical and comprehensive way; combines the genetic algorithm with the scheduling problem; solves the coding problem of scheduling; designs a simple and effective fitness function; and obtains the influence of factors such as population size, crossover probability, and variation probability on scheduling through scheduling simulation experiments; finally, experiments are obtained to find an optimal solution when converging and iterations are obtained. As a result, the general framework and idea of solving the scheduling problem by genetic algorithm are proposed, and an effective solution to the scheduling problem in university is obtained. The test results show that the algorithm can solve the class scheduling problem efficiently and reasonably, and it can eliminate all the resource conflicts and meet the scheduling constraints set by the faculty to the maximum extent without considering the class setting errors. This paper also designs an English social platform based on genetic algorithm to analyze the mode of teaching English classroom in university. In future research, if the college English teaching mode is dynamically improved and the latest teaching status is followed up in real time, this is the future research direction and research hotspot.

## Data Availability

The data used to support the findings of this study are available from the corresponding author upon request.

## References

[1] Wang T. P. (2007). The comparison of the difficulties between cooperative learning and traditional teaching methods in college English teachers. *The Journal of Human Resource and Adult Learning*.

[2] Elizabeth M. E. S. (2010). *Methods of Teaching English*.

[3] Smagorinsky P., Whiting M. E. (1995). *How English Teachers Get Taught: Methods of Teaching the Methods Course*.

[4] Khasawneh M. (2022). The relationship of curriculum, teaching methods, assessment methods, and school and home environment with learning difficulties in English language from the studetns’ perspectives. *Journal of Innovation in Educational and Cultural Research*.

[5] Jerrim J., Vignoles A. (2016). The link between East Asian ’mastery’ teaching methods and English children’s mathematics skills. *Economics of Education Review*.

[6] Songbatumis A. M. (2017). Challenges in teaching English faced by English teachers at MTsN Taliwang, Indonesia. *Journal of foreign language teaching and learning*.

[7] Ghazali S. N., Setia R., Muthusamy C. (2009). ESL students’ attitude towards texts and teaching methods used in literature classes. *English Language Teaching*.

[8] Wang X. (2016). Discussion on application of multimedia teaching in college English vocabulary teaching. *Open Journal of Modern Linguistics*.

[9] Intarapanich C. (2013). Teaching methods, approaches and strategies found in efl classrooms: a case study in Lao pdr. *Procedia - Social and Behavioral Sciences*.

[10] Erton I. (2006). Semiotic nature of language teaching methods in foreign language learning and teaching. *Journal of Language and Linguistic Studies*.

[11] Sun Z., Anbarasan M., Praveen Kumar D. (2021). Design of online intelligent English teaching platform based on artificial intelligence techniques. *Computational Intelligence*.

[12] Zubkov A. D. MOOCs in Blended English Teaching and Learning for Students of Technical Curricula.

[13] Zhang Y. (2021). The development of an evaluation model to assess the effect of online English teaching based on fuzzy mathematics. *International Journal of Emerging Technologies in Learning (iJET)*.

[14] Babinčáková M., Bernard P. (2020). Online experimentation during COVID-19 secondary school closures: teaching methods and student perceptions. *Journal of Chemical Education*.

[15] Morgan H. (2015). Online instruction and virtual schools for Middle and high school students: twenty-first-century fads or progressive teaching methods for today’s pupils?. *The Clearing House: A Journal of Educational Strategies, Issues and Ideas*.

[16] Mbukusa N. R. (2018). Perceptions of students’ on the use of WhatsApp in teaching methods of English as second language at the university of Namibia. *Journal of Curriculum and Teaching*.

[17] Mirjalili S. (2019). Genetic algorithm. *Studies in Computational Intelligence*.

[18] Katoch S., Chauhan S. S., Kumar V. (2021). A review on genetic algorithm: past, present, and future. *Multimedia Tools and Applications*.

[19] Wang Y. H., Li Y. C., Liao H. C. (2011). Using a genetic algorithm to determine optimal complementary learning clusters for ESL in Taiwan. *Expert Systems with Applications*.

[20] Xu J. (2021). Improved Genetic Algorithm to Solve the Scheduling Problem of College English Courses. *Complexity*.

[21] Zhike Y. (2013). A new BP neural network algorithm and its application in English education evaluation. *TELKOMNIKA Indonesian Journal of Electrical Engineering*.

